# Spontaneous Healing of a Rectovaginal Fistula Developing after Laparoscopic Segmental Bowel Resection for Intestinal Deep Infiltrating Endometriosis

**DOI:** 10.1155/2013/837903

**Published:** 2013-04-27

**Authors:** William Kondo, Reitan Ribeiro, Carlos Henrique Trippia, Monica Tessmann Zomer

**Affiliations:** ^1^Department of Gynecology, Sugisawa Medical Center, Avenida Getulio Vargas, 3163 Ap. 21, 80240-041 Curitiba, PR, Brazil; ^2^Department of Radiology, Roentgen Diagnóstico Institute, Curitiba, PR, Brazil

## Abstract

The surgical treatment of intestinal deep infiltrating endometriosis has an associated risk of major complications such as dehiscence of the intestinal anastomosis, pelvic abscess, and rectovaginal fistula. The management of postoperative rectovaginal fistula frequently requires a reoperation and the construction of a stoma for temporary fecal diversion. In this paper we describe a 27-year-old woman undergoing laparoscopic treatment of deep infiltrating endometriosis (extramucosal cystectomy, resection of the uterosacral ligaments, resection of the posterior vaginal fornix, and segmental bowel resection) complicated by a rectovaginal fistula, which healed spontaneously with nonsurgical conservative treatment.

## 1. Introduction

Intestinal deep infiltrating endometriosis (DIE) is defined as lesions involving at least the muscularis propria of the bowel [1]. It affects up to 45.4% of women with DIE lesions [2, 3] and may be found at any level from the anal verge to the small intestine; however, the most frequent sites of involvement are the rectum and the sigmoid colon [3–6]. 

The management of intestinal DIE lesions may be medical and/or surgical. Medical treatment plays a substantial role in terms of pain relief in women with rectovaginal endometriosis; however it has a temporary effect [7]. Surgical treatment combines different procedures according to the anatomical distribution of the lesions [1, 3]. There are different surgical techniques to approach the intestinal DIE: rectal shaving, mucosal skinning, disc resection, and segmental bowel resection (with or without protective ileostomy). The choice for the procedure depends on several factors, such as (1) size of the lesion, (2) percentage of the circumference of the bowel involved by the lesion, (3) presence of multifocal lesions, and (4) distance between the anal verge and the intestinal DIE lesion [8–11]. 

No matter which type of rectal surgery is selected, there is an associated risk of major complications such as dehiscence of the intestinal anastomosis, pelvic abscess, and rectovaginal fistula [12–16]. The latter is a catastrophic complication of such intervention because it may drastically alter patient's self-esteem and intimate relationships and may lead to significant psychosocial and sexual dysfunction [17, 18]. The management of this situation is not easy and frequently requires a reoperation and the construction of a stoma for temporary fecal diversion. 

The aim of this paper is to report one case of spontaneous healing of a rectovaginal fistula developing after laparoscopic segmental bowel resection for intestinal DIE lesion. 

## 2. Case Presentation

A 27-year-old woman came to our service complaining about dysmenorrhea, dyspareunia, chronic pelvic pain, cyclic digestive symptoms (tenesmus and diarrhea during menses), and cyclic urinary symptoms (polacyuria during menses). At rectovaginal examination, a firm 30 mm nodule was palpable at the retrocervical area. 

Transvaginal ultrasound demonstrated a large irregular and retractile plaque-like lesion located at the retrocervical area measuring 40 mm in diameter, involving the serosa of the cervix, the posterior vaginal fornix, both uterosacral ligaments and the anterior rectal wall, around 6 cm above the anal verge (infiltrating up to the submucosa and involving 40% of the circumference of the rectum). Another irregular and retractile lesion was observed at the vesicouterine fold, adherent to the posterior-superior aspect of the bladder, measuring 15 mm in diameter.

**Figure 1:**
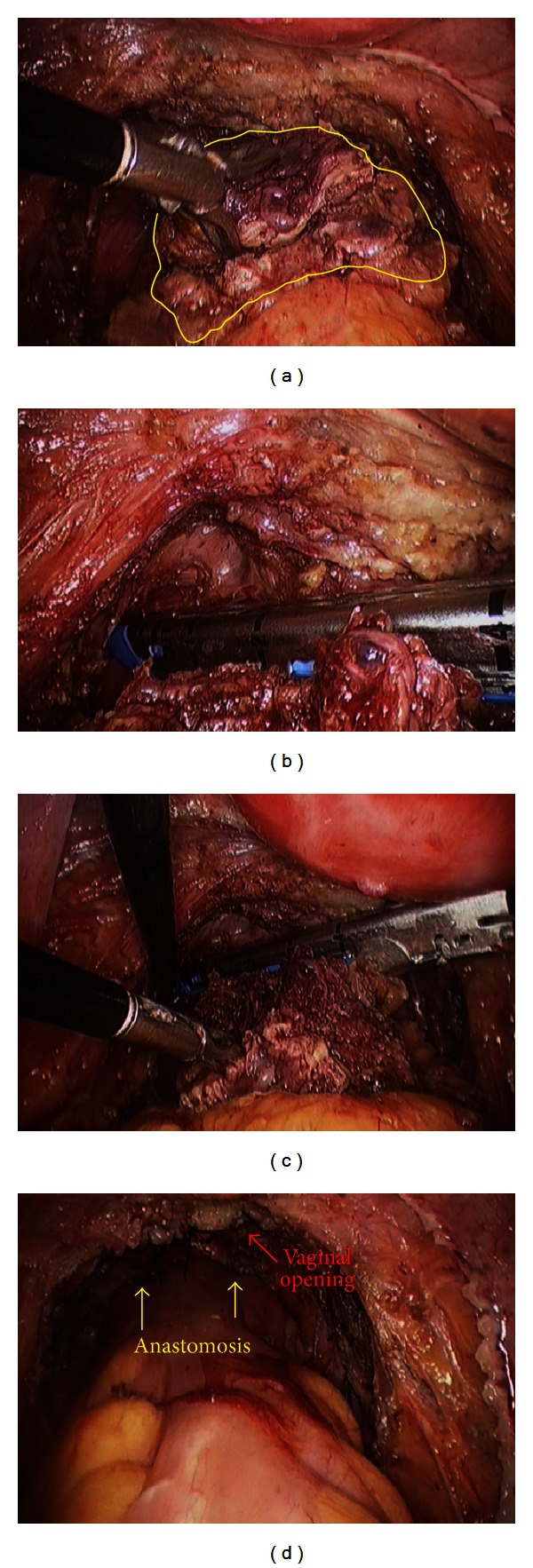
(a) Endometriotic nodule infiltrating the anterior rectal wall. (b and c) Placement of the laparoscopic linear cutting stapler at the rectum. (d) Final view of the procedure including the colorectal anastomosis and the vaginal suture repairing the colpectomy.

After preoperative counseling and signature of the preoperative informed consent form, she underwent laparoscopic treatment of deep infiltrating endometriosis including extramucosal cystectomy, resection of the uterosacral ligaments, resection of the posterior vaginal fornix, and segmental bowel resection. The colorectal anastomosis was placed 5 cm from the anal verge (Figure 1).

She started receiving clear liquids in the first postoperative day and she was discharged home in the second postoperative day with the Foley catheter in place. On the postoperative day 5 she started complaining about malodorous vaginal discharge. She came back to our office and on vaginal examination we could identify a 5 mm hole in the posterior vaginal wall with fecal discharge coming from it. She was hemodynamically stable, with no signs of peritonitis, no fever, and no abdominal pain. Transabdominal and transvaginal ultrasound demonstrated no presence of free intraperitoneal fluid. There was no leukocytosis on blood tests. She was admitted to the hospital again and put on a dietary restriction (high-absorption diet). Blood tests for infection (white blood cell count, erythrocyte sedimentation rate, and C-reactive protein) were collected every day and there was no significant modification on the laboratory exams during 3 days of observation. The Foley catheter was removed on the postoperative day 7 and she was discharged home on the fourth day of readmission. 

She experienced a progressive reduction in the vaginal discharge. Seven days after the onset of the rectovaginal fistula she started passing stools per anus and the vaginal leakage completely stopped 12 days after the onset of the complication. Pelvic CT scan on days 15 and 30 did not show any pelvic fluid collection. Postoperative evaluations on days 30, 60, and 90 did not reveal any evidence of vaginal discharge. 

**Figure 2:**
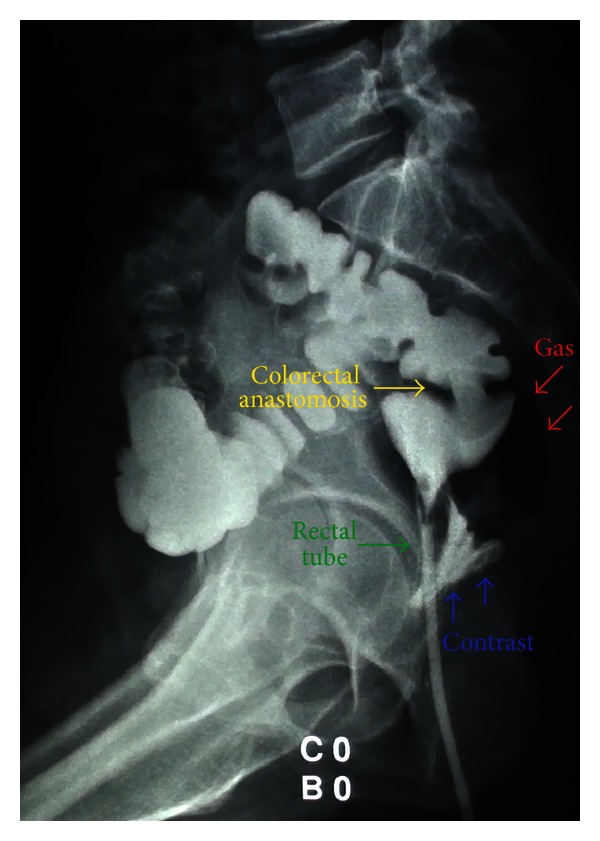
Barium enema did not reveal any evidence of rectovaginal fistula. There was a small amount of gas in the presacral space and contrast material at the posterior pouch of Douglas, with no clinical significance because the patient was asymptomatic.

Barium enema on day 90 did not show any evidence of rectovaginal fistula (Figure 2). After that she was allowed to have sexual intercourse with her husband. 

## 3. Discussion

A fistula is an abnormal communication between 2 epithelialized surfaces. Rectovaginal fistula is a medical condition in which there is an abnormal connection between the rectum and the vagina. Most rectovaginal fistulae arise from obstetric and vaginal trauma; however, inflammatory bowel disease, radiation proctitis, cancer, pelvic infection, and surgery are other causes [18]. 

The two major complications of the surgical treatment of intestinal DIE are anastomotic leakage and rectovaginal fistula [15, 16, 19–21]. Anastomotic dehiscence and leakage seem to occur after segmental bowel resection in 3% to 7% of cases and up to 20% in low rectal anastomosis [22, 23]. An independent predictor for postoperative anastomotic leaks after segmental bowel resection is the colorectal anastomosis less than 10 cm from the anal verge [24–27]. That is why temporary diverting ileostomy seems to be advisable in such cases [24, 27]. The use of the double-stapled technique, and combined resection of the uterus and/or partial vaginectomy during proctectomy are also identified as risk factors for the development of rectovaginal fistula [18]. In the systematic review conducted by Meuleman et al. [19] including 2036 women undergoing segmental bowel resection for intestinal DIE the rates of rectovaginal fistula and anastomotic leakage were 2.7% and 1.5%, respectively. In the large series of Ruffo et al. [16], laparoscopic resection of the mid/low rectum for deep infiltrating endometriosis was conducted in 750 women and the rates of anastomotic leak and rectovaginal fistula were 3% and 2%, respectively. Temporary ileostomy rate was 14.5%.

Rectovaginal fistulae can be classified into two types: low and high varieties. Low rectovaginal fistula is located between the lower third of the rectum and the lower half of the vagina. A high fistula is located between the middle third of the rectum and the posterior vaginal fornix. Small-sized fistulas are less than 0.5 cm in diameter, medium-sized fistulas are 0.5–2.5 cm and large-sized fistulas exceed 2.5 cm [17].

Surgery remains the mainstay of rectovaginal fistula treatment and proper timing is essential in planning an operation for it. The surgeon must choose among transanal, perineal, transvaginal, and an abdominal approach. The location, cause, quality of surrounding tissue, history of repair, and degree of incontinence dictate which approach is taken and the success rate of the repair [18]. Recently, encouraging results have been observed using bioprosthetic sheet and bioprosthetic plug for the treatment of rectovaginal fistula [28]. Nevertheless, some cases of spontaneous closure of rectovaginal fistula have already been published in the literature [29, 30].

In the case presented here, the patient developed a small-sized rectovaginal fistula in the fifth postoperative day. She was investigated for signs of infection on laboratory exams and for any evidence of intra-abdominal fluid collection on imaging exams with no positive findings. Serial laboratory and imaging examinations were performed. She was informed about the need for a reoperation and the construction of a temporary diverting ileostomy. Alternatively, an expectant management was proposed, considering that she did not present neither abdominal pain nor any signs of infection, in order to wait for the best timing to try a conservative treatment with bioprosthetic plug later. Surprisingly, the vaginal leakage progressively reduced and the rectovaginal fistula closed spontaneously. 

Retrospectively evaluating our case, we can observe that the barium enema shows gas in the presacral space as well as contrast material at the posterior pouch of Douglas. Therefore, one explanation for the spontaneous closure of the fistula is that the anastomotic dehiscence occurred at the posterior aspect of the colorectal anastomosis and the fecal material drained through the posterior vaginal fornix. As there was no direct contact between the orifice of the anastomotic dehiscence and the vaginal orifice, the small anastomotic dehiscence closed spontaneously with dietary restriction followed by the healing of the posterior vaginal fornix. Indeed, this is not the standard way to treat a rectovaginal fistula but was an option in this specific case due to the absence of signs of infection.
